# Supplementary material for: Multi‐dataset identification of innovative feature genes and molecular mechanisms in keratoconus
Source: J Cell Mol Med. 2024 Sep 19;28(18):e70079. doi: 10.1111/jcmm.70079 (PMC11412914; doi:10.1111/jcmm.70079)
Supplement: Supplementary file 2 — Material S2. [file JCMM-28-e70079-s002.zip › 7.GSEA analysis/4.MYRF/KEGG_GLYCOSAMINOGLYCAN_DEGRADATION.html]

Details for gene set KEGG\_GLYCOSAMINOGLYCAN\_DEGRADATION[GSEA]

|  || Dataset | MYRF.group.cls#h\_versus\_l |
| Phenotype | group.cls#h\_versus\_l |
| Upregulated in class | h |
| GeneSet | KEGG\_GLYCOSAMINOGLYCAN\_DEGRADATION |
| Enrichment Score (ES) | 0.29915407 |
| Normalized Enrichment Score (NES) | 0.94531816 |
| Nominal p-value | 0.49266246 |
| FDR q-value | 1.0 |
| FWER p-Value | 1.0 |
Table: GSEA Results Summary

  

Fig 1: Enrichment plot: KEGG\_GLYCOSAMINOGLYCAN\_DEGRADATION      
 Profile of the Running ES Score & Positions of GeneSet Members on the Rank Ordered List

  

| SYMBOL | TITLE | RANK IN GENE LIST | RANK METRIC SCORE | RUNNING ES | CORE ENRICHMENT || 1 | HS3ST3A1 | na | 567 | 0.556 | 0.2562 | Yes |
| 2 | ARSB | na | 3619 | 0.267 | 0.2992 | Yes |
| 3 | HS3ST3B1 | na | 7349 | 0.154 | 0.2674 | No |
| 4 | HYAL3 | na | 12363 | 0.058 | 0.1517 | No |
| 5 | HYAL4 | na | 12568 | 0.056 | 0.1731 | No |
| 6 | HYAL1 | na | 12628 | 0.055 | 0.1984 | No |
| 7 | NAGLU | na | 12941 | 0.051 | 0.2144 | No |
| 8 | HGSNAT | na | 14222 | 0.035 | 0.1948 | No |
| 9 | SGSH | na | 14538 | 0.032 | 0.2015 | No |
| 10 | GNS | na | 16172 | 0.016 | 0.1622 | No |
| 11 | HYAL2 | na | 16797 | 0.010 | 0.1493 | No |
| 12 | GUSB | na | 18367 | -0.004 | 0.1060 | No |
| 13 | IDUA | na | 18562 | -0.006 | 0.1033 | No |
| 14 | HEXA | na | 18816 | -0.008 | 0.1001 | No |
| 15 | GALNS | na | 20034 | -0.022 | 0.0759 | No |
| 16 | HPSE | na | 20244 | -0.025 | 0.0820 | No |
| 17 | GLB1 | na | 22760 | -0.056 | 0.0370 | No |
| 18 | HEXB | na | 22837 | -0.057 | 0.0628 | No |
| 19 | HPSE2 | na | 27852 | -0.157 | -0.0049 | No |
| 20 | IDS | na | 29263 | -0.199 | 0.0521 | No |
| 21 | SPAM1 | na | 29743 | -0.215 | 0.1436 | No |
Table: GSEA details [plain text format]

  

Fig 2: KEGG\_GLYCOSAMINOGLYCAN\_DEGRADATION      
 Blue-Pink O' Gram in the Space of the Analyzed GeneSet

  

Fig 3: KEGG\_GLYCOSAMINOGLYCAN\_DEGRADATION: Random ES distribution      
 Gene set null distribution of ES for **KEGG\_GLYCOSAMINOGLYCAN\_DEGRADATION**
